# Quinoxaline-2-carbonitrile

**DOI:** 10.1107/S1600536809051289

**Published:** 2009-12-04

**Authors:** Hoong-Kun Fun, Ching Kheng Quah, Annada C. Maity, Nirmal Kumar Das, Shyamaprosad Goswami

**Affiliations:** a X-ray Crystallography Unit, School of Physics, Universiti Sains Malaysia, 11800 USM, Penang, Malaysia; b Department of Chemistry, Bengal Engineering and Science University, Shibpur, Howrah 711 103, India

## Abstract

In the title compound, C_9_H_5_N_3_, the quinoxaline ring is essentially planar, with a maximum deviation of 0.012 (1) Å. Short inter­molecular distances between the centroids of the 2,3-dihydro­pyrazine and benzene rings [3.6490 (5) Å] indicate the existence of π⋯π inter­actions. In the crystal packing, the mol­ecules are linked *via* two pairs of inter­molecular C—H⋯N inter­actions, forming *R* ^2^_2_ (8) and *R* ^2^_2_ (10) ring motifs; these mol­ecules are further linked into a two-dimensional network parallel to (1 0 2) *via* another C–H⋯N inter­action.

## Related literature

For the synthesis of cyano *N*-heterocyclic compounds, see: Goswami *et al.* (2007 ▶, 2009 ▶). For reference bond lengths, see: Allen *et al.* (1987 ▶). For hydrogen-bond motifs, see: Bernstein *et al.* (1995 ▶). For the stability of the temperature controller used for the data collection, see: Cosier & Glazer (1986 ▶).
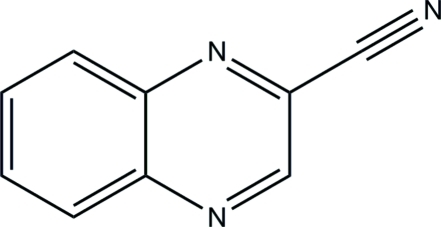


## Experimental

### 

#### Crystal data

C_9_H_5_N_3_
*M* *_r_* = 155.16
Monoclinic, 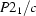

*a* = 3.8055 (1) Å
*b* = 19.0466 (4) Å
*c* = 10.1845 (2) Å
β = 93.466 (1)°
*V* = 736.84 (3) Å^3^
*Z* = 4
Mo *K*α radiation
μ = 0.09 mm^−1^
*T* = 100 K
0.39 × 0.28 × 0.25 mm

#### Data collection

Bruker SMART APEXII CCD area-detector diffractometer
Absorption correction: multi-scan (*SADABS*; Bruker, 2005 ▶) *T*_min_ = 0.966, *T*_max_ = 0.978
11604 measured reflections
2716 independent reflections
2183 reflections with *I* > 2σ(*I*)
*R*_int_ = 0.023

#### Refinement

*R*[*F* ^2^ > 2σ(*F* ^2^)] = 0.047
*wR*(*F* ^2^) = 0.135
*S* = 1.08
2716 reflections
129 parameters
All H-atom parameters refined
Δρ_max_ = 0.53 e Å^−3^
Δρ_min_ = −0.23 e Å^−3^

### 

Data collection: *APEX2* (Bruker, 2005 ▶); cell refinement: *SAINT* (Bruker, 2005 ▶); data reduction: *SAINT*; program(s) used to solve structure: *SHELXTL* (Sheldrick, 2008 ▶); program(s) used to refine structure: *SHELXTL*; molecular graphics: *SHELXTL*; software used to prepare material for publication: *SHELXTL* and *PLATON* (Spek, 2009 ▶).

## Supplementary Material

Crystal structure: contains datablocks global, I. DOI: 10.1107/S1600536809051289/sj2699sup1.cif

Structure factors: contains datablocks I. DOI: 10.1107/S1600536809051289/sj2699Isup2.hkl

Additional supplementary materials:  crystallographic information; 3D view; checkCIF report

## Figures and Tables

**Table 1:** Hydrogen-bond geometry (Å, °)

*D*—H⋯*A*	*D*—H	H⋯*A*	*D*⋯*A*	*D*—H⋯*A*
C2—H2⋯N1^i^	0.984 (14)	2.619 (14)	3.5730 (12)	163.4 (12)
C4—H4⋯N2^ii^	0.988 (13)	2.593 (13)	3.4268 (12)	142.0 (10)
C7—H7⋯N3^iii^	0.998 (14)	2.540 (15)	3.5225 (12)	168.3 (12)

Symmetry codes: (i) 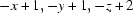; (ii) 
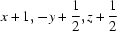
; (iii) 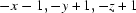.

**Fig. 1.**
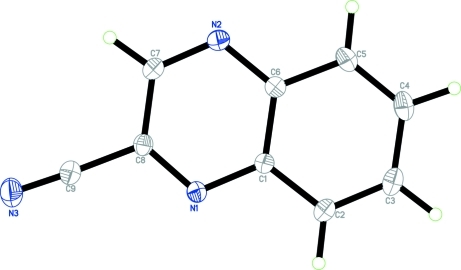
The molecular structure of the title compound, showing 50% probability displacement ellipsoids for non-H atoms and the atom-numbering scheme.

**Fig. 2.**
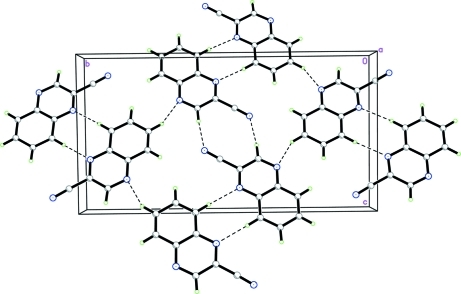
The crystal structure of the title compound viewed along the a axis. Intermolecular interactions are shown in dashed lines.

## supplementary crystallographic information

### Comment

Heterocyclic molecules containing a cyano group are useful as drug intermediates. The development of new pathways leading to efficient synthesis of heterocycles with diversity in skeleton and functional groups is an important field of research in both synthetic and medicinal chemistry. Recently, we have synthesized a number of cyano N-heterocyclic compounds using triselenium dicyanide (TSD) (Goswami *et al.*, 2007, 2009). Herein we report the synthesis of 2-cyanoquinoxaline from quinoxaline under microwave irradiation and its molecular structure.

The bond lengths (Allen *et al.*, 1987) and angles in the title compound (Fig. 1) are within normal ranges. The quinoxaline ring (N1/N2/C1-C8) is essentially planar, with the maximum deviation of 0.012 (1) Å for atom C5. Short intermolecular distances between the centroids of the pyrazine (N1/N2/C1/C6-C8) and benzene rings (C1-C6) [3.6490 (5) Å] indicate the existence of π···π interactions.

In the crystal packing (Fig. 2), the molecules are linked *via* pairs of intermolecular C2—H2···N1 and C7—H7···N3 interactions, forming *R*^2^_2_ (8) and *R*^2^_2_ (10) ring motifs (Bernstein *et al.*, 1995) and these molecules are further linked into two-dimensional networks parallel to plane (1 0 2) *via* C4–H4···N2 interactions.

### Experimental

A thoroughly ground mixture of selenium dioxide (1.32 g, 12 mmol) and malononitrile (0.26 g, 4 mmol) in 4-5 drops of DMSO was kept stirring in an open-mouth conical flask. The mixture became reddish-brown after 7 min. An exothermic reaction began in the next 10 minutes when triselenium dicyanide was formed. The heterocyclic substrate quinoxaline (0.39 g, 3 mmol) was added to the mixture after the termination of the exothermic reaction. The conical flask was placed in a domestic microwave oven at 240 W for 20 min. The progress of the reaction was monitored by TLC. After completion of the reaction, water was added and the mixture was extracted with chloroform. The organic layer was washed with saturated brine and dried over MgSO_4_ and followed by evaporation with a rotary evaporator under low pressure to afford a light yellow substance. This was purified on silica gel (60-120 mesh) column chromatography eluting with petroleum ether (boiling point, 60-80° C) to give the compound (0.34 g, 74 %) as a crystalline solid.

### Refinement

All H atoms were located in a difference Fourier map and refined freely.

### Crystal data

**Table:**

C_9_H_5_N_3_	*F*(000) = 320
*M**_r_* = 155.16	*D*_x_ = 1.399 Mg m^−^^3^
Monoclinic, *P*2_1_/*c*	Mo *K*α radiation, λ = 0.71073 Å
Hall symbol: -P 2ybc	Cell parameters from 4710 reflections
*a* = 3.8055 (1) Å	θ = 2.9–32.7°
*b* = 19.0466 (4) Å	µ = 0.09 mm^−^^1^
*c* = 10.1845 (2) Å	*T* = 100 K
β = 93.466 (1)°	Block, yellow
*V* = 736.84 (3) Å^3^	0.39 × 0.28 × 0.25 mm
*Z* = 4	

### Data collection

**Table:**

Bruker SMART APEXII CCD area-detector diffractometer	2716 independent reflections
Radiation source: fine-focus sealed tube	2183 reflections with *I* > 2σ(*I*)
graphite	*R*_int_ = 0.023
φ and ω scans	θ_max_ = 32.8°, θ_min_ = 2.1°
Absorption correction: multi-scan (*SADABS*; Bruker, 2005)	*h* = −5→5
*T*_min_ = 0.966, *T*_max_ = 0.978	*k* = −29→21
11604 measured reflections	*l* = −15→14

### Refinement

**Table:**

Refinement on *F*^2^	Primary atom site location: structure-invariant direct methods
Least-squares matrix: full	Secondary atom site location: difference Fourier map
*R*[*F*^2^ > 2σ(*F*^2^)] = 0.047	Hydrogen site location: inferred from neighbouring sites
*wR*(*F*^2^) = 0.135	All H-atom parameters refined
*S* = 1.08	*w* = 1/[σ^2^(*F*_o_^2^) + (0.0782*P*)^2^ + 0.0918*P*] where *P* = (*F*_o_^2^ + 2*F*_c_^2^)/3
2716 reflections	(Δ/σ)_max_ = 0.001
129 parameters	Δρ_max_ = 0.53 e Å^−^^3^
0 restraints	Δρ_min_ = −0.23 e Å^−^^3^

### Special details

**Table:**

Experimental. The crystal was placed in the cold stream of an Oxford Cyrosystems Cobra open-flow nitrogen cryostat (Cosier & Glazer, 1986) operating at 100.0 (1) K.
Geometry. All esds (except the esd in the dihedral angle between two l.s. planes) are estimated using the full covariance matrix. The cell esds are taken into account individually in the estimation of esds in distances, angles and torsion angles; correlations between esds in cell parameters are only used when they are defined by crystal symmetry. An approximate (isotropic) treatment of cell esds is used for estimating esds involving l.s. planes.
Refinement. Refinement of F^2^ against ALL reflections. The weighted R-factor wR and goodness of fit S are based on F^2^, conventional R-factors R are based on F, with F set to zero for negative F^2^. The threshold expression of F^2^ > 2sigma(F^2^) is used only for calculating R-factors(gt) etc. and is not relevant to the choice of reflections for refinement. R-factors based on F^2^ are statistically about twice as large as those based on F, and R- factors based on ALL data will be even larger.

### Fractional atomic coordinates and isotropic or equivalent isotropic displacement parameters (Å^2^)

**Table:**

	*x*	*y*	*z*	*U*_iso_*/*U*_eq_	
N1	0.12156 (18)	0.46098 (4)	0.84114 (7)	0.01481 (16)	
N2	−0.12366 (19)	0.33470 (4)	0.71846 (8)	0.01659 (17)	
N3	−0.2187 (2)	0.57757 (5)	0.60971 (9)	0.0269 (2)	
C1	0.1884 (2)	0.39728 (4)	0.89844 (8)	0.01344 (17)	
C2	0.3822 (2)	0.39456 (5)	1.02181 (9)	0.01744 (18)	
C3	0.4514 (2)	0.33059 (5)	1.07937 (10)	0.02033 (19)	
C4	0.3352 (2)	0.26756 (5)	1.01678 (10)	0.0210 (2)	
C5	0.1474 (2)	0.26871 (5)	0.89805 (9)	0.01857 (19)	
C6	0.0674 (2)	0.33382 (4)	0.83638 (9)	0.01441 (17)	
C7	−0.1868 (2)	0.39663 (4)	0.66474 (9)	0.01675 (18)	
C8	−0.0633 (2)	0.45941 (4)	0.72702 (8)	0.01496 (17)	
C9	−0.1450 (2)	0.52598 (5)	0.66332 (9)	0.01913 (19)	
H2	0.471 (4)	0.4375 (7)	1.0662 (14)	0.030 (3)*	
H3	0.591 (4)	0.3282 (7)	1.1650 (16)	0.038 (4)*	
H4	0.396 (3)	0.2229 (7)	1.0622 (13)	0.028 (3)*	
H5	0.057 (4)	0.2262 (7)	0.8533 (14)	0.032 (3)*	
H7	−0.328 (4)	0.4008 (7)	0.5793 (14)	0.028 (3)*	

### Atomic displacement parameters (Å^2^)

**Table:**

	*U*^11^	*U*^22^	*U*^33^	*U*^12^	*U*^13^	*U*^23^
N1	0.0154 (3)	0.0145 (3)	0.0146 (3)	0.0003 (2)	0.0014 (2)	0.0010 (2)
N2	0.0168 (3)	0.0173 (3)	0.0157 (4)	−0.0006 (2)	0.0014 (3)	−0.0016 (3)
N3	0.0328 (4)	0.0235 (4)	0.0241 (5)	0.0028 (3)	−0.0002 (3)	0.0057 (3)
C1	0.0128 (3)	0.0147 (3)	0.0130 (4)	0.0002 (2)	0.0019 (3)	0.0004 (3)
C2	0.0157 (4)	0.0218 (4)	0.0147 (4)	−0.0004 (3)	−0.0002 (3)	0.0005 (3)
C3	0.0164 (4)	0.0275 (4)	0.0170 (4)	0.0019 (3)	0.0003 (3)	0.0055 (3)
C4	0.0176 (4)	0.0208 (4)	0.0248 (5)	0.0030 (3)	0.0042 (3)	0.0088 (3)
C5	0.0179 (4)	0.0147 (4)	0.0235 (5)	0.0007 (3)	0.0036 (3)	0.0029 (3)
C6	0.0131 (3)	0.0152 (3)	0.0151 (4)	0.0000 (2)	0.0027 (3)	0.0000 (3)
C7	0.0164 (4)	0.0195 (4)	0.0142 (4)	−0.0003 (3)	0.0000 (3)	−0.0004 (3)
C8	0.0145 (3)	0.0168 (4)	0.0137 (4)	0.0005 (3)	0.0019 (3)	0.0015 (3)
C9	0.0204 (4)	0.0202 (4)	0.0167 (4)	0.0005 (3)	0.0005 (3)	0.0015 (3)

### Geometric parameters (Å, °)

**Table:**

N1—C8	1.3220 (11)	C3—C4	1.4175 (14)
N1—C1	1.3636 (10)	C3—H3	0.995 (16)
N2—C7	1.3161 (11)	C4—C5	1.3670 (13)
N2—C6	1.3659 (11)	C4—H4	0.988 (13)
N3—C9	1.1506 (12)	C5—C6	1.4150 (11)
C1—C2	1.4190 (12)	C5—H5	0.980 (14)
C1—C6	1.4273 (11)	C7—C8	1.4202 (12)
C2—C3	1.3706 (12)	C7—H7	0.997 (14)
C2—H2	0.985 (14)	C8—C9	1.4495 (12)
			
C8—N1—C1	115.57 (7)	C4—C5—C6	119.57 (8)
C7—N2—C6	116.78 (7)	C4—C5—H5	123.2 (8)
N1—C1—C2	119.01 (7)	C6—C5—H5	117.2 (8)
N1—C1—C6	121.14 (8)	N2—C6—C5	119.33 (7)
C2—C1—C6	119.85 (7)	N2—C6—C1	121.28 (7)
C3—C2—C1	119.16 (8)	C5—C6—C1	119.38 (8)
C3—C2—H2	119.4 (8)	N2—C7—C8	121.44 (8)
C1—C2—H2	121.5 (8)	N2—C7—H7	120.7 (7)
C2—C3—C4	120.92 (9)	C8—C7—H7	117.9 (7)
C2—C3—H3	119.6 (8)	N1—C8—C7	123.77 (7)
C4—C3—H3	119.4 (8)	N1—C8—C9	117.52 (7)
C5—C4—C3	121.10 (8)	C7—C8—C9	118.71 (8)
C5—C4—H4	121.5 (8)	N3—C9—C8	177.49 (10)
C3—C4—H4	117.4 (8)		
			
C8—N1—C1—C2	−179.34 (7)	C4—C5—C6—C1	0.84 (13)
C8—N1—C1—C6	0.75 (12)	N1—C1—C6—N2	−0.95 (13)
N1—C1—C2—C3	−179.68 (8)	C2—C1—C6—N2	179.14 (7)
C6—C1—C2—C3	0.24 (13)	N1—C1—C6—C5	178.94 (7)
C1—C2—C3—C4	0.62 (13)	C2—C1—C6—C5	−0.97 (12)
C2—C3—C4—C5	−0.75 (14)	C6—N2—C7—C8	−0.32 (13)
C3—C4—C5—C6	0.00 (14)	C1—N1—C8—C7	−0.38 (12)
C7—N2—C6—C5	−179.20 (7)	C1—N1—C8—C9	179.15 (7)
C7—N2—C6—C1	0.69 (12)	N2—C7—C8—N1	0.18 (14)
C4—C5—C6—N2	−179.26 (7)	N2—C7—C8—C9	−179.36 (8)

### Hydrogen-bond geometry (Å, °)

**Table:**

*D*—H···*A*	*D*—H	H···*A*	*D*···*A*	*D*—H···*A*
C2—H2···N1^i^	0.984 (14)	2.619 (14)	3.5730 (12)	163.4 (12)
C4—H4···N2^ii^	0.988 (13)	2.593 (13)	3.4268 (12)	142.0 (10)
C7—H7···N3^iii^	0.998 (14)	2.540 (15)	3.5225 (12)	168.3 (12)

Symmetry codes: (i) −*x*+1, −*y*+1, −*z*+2; (ii) *x*+1, −*y*+1/2, *z*+1/2; (iii) −*x*−1, −*y*+1, −*z*+1.
